# Cases of Cephalic Version in the Postural Position

**Published:** 1882-03-20

**Authors:** John Havenstein

**Affiliations:** President of the Erie County Medical Society


### CASES OF CEPHALIC VERSION IN THE POSTURAL POSITION.

ADDRESS BY JOHN HAVENSTEIN, M. D.,
President of the Erie County Medical Society. Read at the Annual Meeting January 10, 1882.

CASE I. Mrs. Van V., aged 31 years, tall, with well marked lateral curvature of the spine, was taken with labor pains with her first child at 9 o'clock p. m., on the twentieth of June 1879, and in the absence of Dr. W. Ring, his son, Dr. Chas. A. Ring, attended her during the night. In the morning, Dr. W. Ring called and found the os uteri dilated to the size of a half dollar, the face presenting with the chin toward the left side. At 10 o'clock the following morning I first saw the patient in consultation with Drs. W. and Chas. A. Ring. An examination confirmed their diagnosis, and detected at the same time a marked projection of the sacrum forward, and consequent diminution of the conjugate diameter of the pelvis. The membranes had ruptured and the face was pressed firmly against the brain. It was agreed to attempt version of the head. The woman was placed in the knee and chest position, and I then passed my right hand into the uterus as far as enabled me to grasp the occiput, then flexing the head upon the chest, I succeeded in bringing down the vertex. In consequence, however, of the deformity, the head did not engage in the pelvis, and three hours after version of the head the forceps were applied and the woman delivered of a healthy child. The mother's convalescence was natural, and the child was thrifty in appearance.

CASE II. Dr. W. Ring sent for me at 10 o'clock a. m., December 8, 1880, to assist him in a case of face labor. The woman, Mrs. S., 29 years of age, with her first child, had been in labor twenty-four hours before I saw her. An examination proved the chin to be to left side, and not yet engaged in the upper strait. In consultation it was agreed, in order to expedite the labor and to relieve the woman of her sufferings, to resort to cephalic version. She was placed in the postural position, and my right hand was then passed over the occiput, and the face presentation was converted into one of the vertex. Labor was completed by the natural powers two hours after version. Mother and child made a good recovery, although the mother needed more than ordinary care to guard against inflammation.

CASE III. Mrs. C., aged 35 years, fleshy and strong, commenced her fourth labor at 5 o'clock a. m., March 17, 1881. Dr. Ring was called at 8 o'clock, and found membranes ruptured, the child presenting face downward, chin toward the left sacro-iliac symphysis but more nearly to the sacrum, child's head very large. At 10 o'clock a. m., Dr. Ring sent for me, and upon my arrival I found the condition as stated. The woman had powerful labor pains, and the face of the child was pressed with great violence against the brain of the pelvis, causing it to swell considerably. As soon, however, as the woman was placed in the postural position, my hand could readily be passed into the uterus; I found the fœtal head to be free and very movable, so much so that in order to fix it, I had to grasp the occiput with the points of my fingers. Version being completed, the child was born twenty minutes after; the effects of pressure were shown by a swelled face and closed eyes for several days, consequent upon the strong labor pains. The child weighed twelve pounds. Mother and child made an excellent recovery.

Such being some of my experience in face presentations, I concluded, after some hesitation, to acquaint my professional colleagues of this society with the facts of a mode of procedure in such cases, that I have not found mentioned in the writings ol obstetricians. For any merit that there may be in this method of correcting face presentations, I can truthfully say I am under obligations to no one for hints or suggestions in its adoption. The claim for originality, however, is immaterial; if I have succeeded in calling your attention to a practice which you will recognize and acknowledge after trial as an improvement in the management of face labor, *my labors* are compensated.—*Med. and Sur. Journal.*

